# Design, Characterization, and Antimicrobial Evaluation of Copper Nanoparticles Utilizing Tamarixinin a Ellagitannin from Galls of *Tamarix aphylla*

**DOI:** 10.3390/ph15020216

**Published:** 2022-02-11

**Authors:** Mohamed A. A. Orabi, Mounir M. Salem-Bekhit, Ehab I. Taha, El-Shaymaa Abdel-Sattar, Omaish Salman Alqahtani, Fakhria A. Al-Joufi, Basel A. Abdel-Wahab, Ali Mohamed Alshabi, Hamad S. Alyami, Javed Ahmad, Tsutomu Hatano

**Affiliations:** 1 Department of Pharmacognosy, College of Pharmacy, Najran University, Najran 1988, Najran, Saudi Arabia; osalqahtani@nu.edu.sa; 2 Kyyali Chair for Pharmaceutical Industry, Department of Pharmaceutics, College of Pharmacy, King Saud University, Riyadh 11451, Riyadh, Saudi Arabia; mounirmsalem@yahoo.com; 3 Microbiology and Immunology Department, Faculty of Pharmacy, Al-Azhar University, Cairo 11884, Egypt; 4 Department of Pharmaceutics, College of Pharmacy, King Saud University, P.O. Box 2457, Riyadh 11451, Riyadh, Saudi Arabia; eelbadawi@ksu.edu.sa; 5 Department of Microbiology and Immunology, Faculty of Pharmacy, South Valley University, Qena 83523, Egypt; elshaymaa_a_m@svu.edu.eg; 6 Department of Pharmacology, College of Pharmacy, Jouf University, Sakakah 72341, Al-Jouf, Saudi Arabia; faaljoufi@ju.edu.sa; 7 Department of Pharmacology, College of Pharmacy, Najran University, Najran 1988, Najran, Saudi Arabia; babdelnaem@nu.edu.sa; 8 Department of Clinical Pharmacy, College of Pharmacy, Najran University, Najran 1988, Najran, Saudi Arabia; amalshabi@nu.edu.sa; 9 Department of Pharmaceutics, College of Pharmacy, Najran University, Najran 1988, Najran, Saudi Arabia; jaahmed@nu.edu.sa; 10 Graduate School of Medicine, Dentistry and Pharmaceutical Sciences, Okayama University, Tsushima, Okayama 700-8530, Japan; hatano-t@cc.okayama-u.ac.jp

**Keywords:** copper nanoparticles, green synthesis, antimicrobial activity, ellagitannins, tamarixinin A

## Abstract

The application of plant extracts or plant-derived compounds in the green synthesis of metal nanoparticles (NPs) was researched. Determining the exact metabolite implicated in the formation of NPs would necessitate comprehensive investigations. Copper nanoparticles (CuNPs) are gaining a lot of attention because of their unique properties and effectiveness against a wide range of bacteria and fungi, as well as their potential for usage in catalytic, optical, electrical, and microelectronics applications. In the course of this study, we aimed to formulate CuNPs utilizing pure tamarixinin A (TA) ellagitannin isolated from *Tamarix aphylla* galls. The main particle size of the formed CuNPs was 44 ± 1.7 nm with zeta potential equal to −23.7 mV, which emphasize the stability of the CuNPs. The X-ray diffraction spectroscopy showed a typical centered cubic crystalline structure phase of copper. Scanning electron microscopy images were found to be relatively spherical and homogeneous in shape. The antimicrobial properties of TA, as well as its mediated CuNPs, have been evaluated through well diffusion assays against four bacterial, *Bacillus subtilis* NCTC 10400, *Staphylococcus aureus* ATCC 25923, *Escherichia coli* ATCC 25922, and *Pseudomonas aeruginosa* ATCC 27853, and two fungal, *Candida albicans* and *Aspergillus flavus*, strains. The distinctive antimicrobial activities were noted against the fungal strains and the Gram-negative bacterial strains *P. aeruginosa* ATCC 27853, and *E. coli* ATCC 25922. In conclusion, CuNPs mediated by TA can be applied for combating a wide range of bacterial and fungal species especially *C. albicans*, *Asp. flavus*, and *P. aeruginosa* in a variety of fields.

## 1. Introduction

Nanotechnology is gaining substantial momentum these days owing to remarkably enhanced in vivo fate and subsequently therapeutic efficacy, reduced dose and dosing frequency, and significantly mitigated toxicity profile of incorporated active ingredient in nanoformulation. Metal nanoparticles (NPs) including gold, silver, iron, and copper nanoparticles (CuNPs) have also been established as potent and efficacious nanoformulation strategies for a diverse variety of indications, particularly microbial infections [1,2,3,4]. Random and/or improper use of antibacterial drugs has resulted in the development of microbial resistance to a range of antimicrobials. Therefore metal NPs have emerged as a possibility for combating these resistant microbes [5]. CuNPs have been widely explored for their antimicrobial potentials in a plethora of research studies, with the results demonstrating that they are effective against a wide range of bacterial and fungal strains [5,6]. Following preceding literature, the antimicrobial effect of CuNPs could be attributed to their small size, and high surface to volume ratio, which allows them to intermingle with microbial cell membranes appropriately and intimately and exert their microbicide effect [1].

The major drawback with the chemical synthesis of metal NPs was the use of hazardous and expensive chemicals in their fabrication process that raise in vivo toxicity and environmental pollution concerns. To address this issue, novel and natural processing agents have been investigated in the present research work that is completely eco-friendly, non-toxic, and has tremendous potential of expediting the process of production of metal NPs [1]. The implication of natural reducing agent for NPs formation resolve environmental and toxicity concerns and is appreciated from the perspective of green chemistry. Many studies have demonstrated the use of the green synthesis technique to manufacture metal nanoparticles using extracts from various plant organs, such as the seed, stem, flower, leaf, and skin of the fruits [7,8,9]. Plant extracts provide potential alternatives that can serve as both stabilizing and reducing agents [10]. In addition, the nanoparticles mediated by natural reductant were found to be associated with the medicinal properties of that agent, which could be exploited in medication, targeted drug delivery, and other purposes. Polyphenols including flavonoids and tannins are among the phytochemicals found in plants that help produce metallic NPs by reducing their original salts and stabilizing the obtained NPs in an oxidation–reduction-mediated process. In addition, there have been a multitude of options of tannin-rich plant extracts (e.g., chestnut, mangrove and quebracho) that can be successfully utilized in the green production of metal NPs [11]. However, the process of formulation of metal NPs employing plant extracts is complex due to numerous obstacles in determining the specific chemical components involved in the creation of metallic NPs, in addition to the effort required to purify the produced NPs from the remaining plant extract [12]. Purified tannic acid, a gallotannin with core glucose and ten galloyl groups or more, has been demonstrated to be an effective reducing agent, stabilizer, and surface modifier for the development of various metal NPs [13,14].

**Figure 1:**
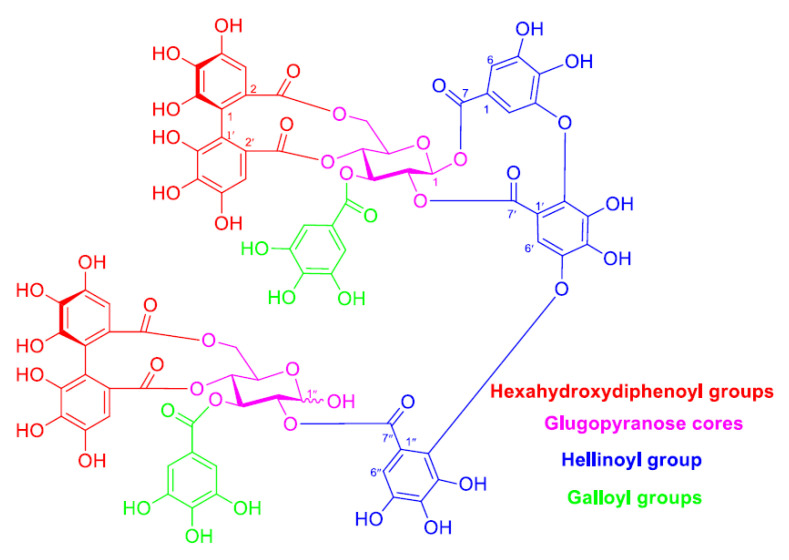
Molecular structure of tamarixinin A.

In our preceding research publications, tannin-rich plant sources including galls of *T. aphylla* were deciphered [15] which yields approximately 71% total extract, with hydrolyzable tannins accounting for the majority [15,16]. The highest occurring type of ellagitannins in the galls of *T. aphylla*, according to our previous research, are those having a sugar core with a free hydroxyl group at the C-1 position, wherein the ellagitannin tamarixinin A (TA) is the most prevalent one [17]. TA is an ellagitannin dimer (Figure 1) with a wide range of reducing functionalities (OH, CO, and C-O-C), making it an excellent agent for reducing copper ions to metal CuNPs. TA was also found to be a major ellagitannin in various Tamaricaceae plants including *T. nilotica*, *T. tetrandra*, and *Reaumuria hirtella*, which are common invasive plants in many deserts and Oases of the Middle East [18,19,20,21].

In the present study, TA was explored as part of a green chemistry approach for the synthesis of CuNPs. The prepared CuNPs were evaluated for physico-chemical characterization including assessment of morphology and particle diameter by scanning electron microscopy (SEM). To confirm the authenticity and crystalline nature of synthesized CuNPs, characterization with UV-Visible spectroscopy and X-ray Diffraction (XRD) was employed, respectively. Subsequently, the synthesized CuNPs, as well as the TA, were evaluated for their antibacterial and antifungal efficacy.

## 2. Results and Discussion

### 2.1. Structural Feature of TA

In the present study, TA was obtained as the most abundant ellagitannin from the aqueous acetone extract of *T. aphylla* galls. TA was obtained in high purity (Figure S1) after repeated chromatographic purifications of the extract (the procedure was given in the experimental section). Its structure was identified by comparing its ^1^H NMR data with that of TA from our earlier research publication on the tannin of *T. nilotica* [19]. TA is an ellagitannin dimer composed of two HHDP moieties, two unsubstituted galloyls, a hellinoyl moiety, and two glucose cores (one of them is presenting a non-bonded hydroxyl group at position 1) (Figure 1). It has a lot of reducing functionalities (OH, CO, and C-O-C), which makes it an excellent agent for the formulation, capping, and stabilization of metal NPs. Investigation of ellagitannins-rich crude extracts indicated the involvement of the same groups in the reduction and stabilization of the metal nanoparticles [11].

### 2.2. Characterization of CuNPs

#### 2.2.1. Visual Observation

Visual inspection of the color change in the reactant’s mixture is a good sign of the ongoing process of formation of CuNPs. Changing the color from blue before the addition of TA to dull bluish brown and finally to dark green is very useful as conclusive criteria [22].

#### 2.2.2. UV-Visible Spectroscopy

**Figure 2:**
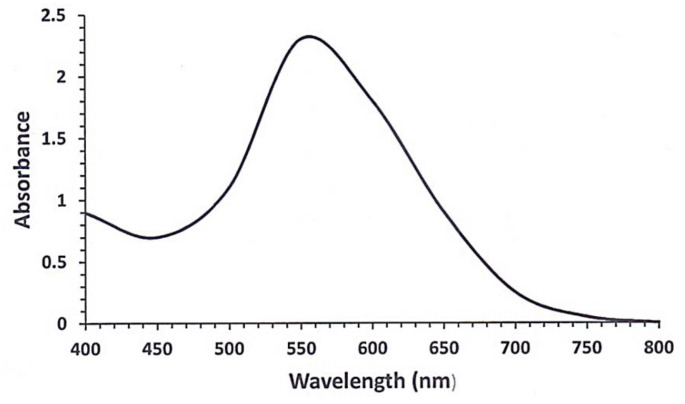
Visible spectrum of TA-mediated CuNPs.

Monitoring the UV-Visible spectrum has been implicated in following the reduction of copper sulfate into copper metal [22]. CuNPs display a surface plasmon peak (absorption bands) in the region from 550 to 700 nm. Copper sulfate solution changed from a dull bluish brown color to a dark green color after TA was added, which showed the UV-Visible spectrum of CuNPs surface plasmon peak at 553 nm (Figure 2). As per literature reports, CuNPs were synthesized using *Fortunella margarita* leaf extract with corroborating results [22].

#### 2.2.3. Particle Size, Zeta Potential, and Polydispersity Index Evaluation

Particle size analysis confirms the formation of CuNPs. CuNPs was found to be in the nanometer range (44 ± 1.7 nm) which indicates the efficiency of TA as a reducing agent. The low value of polydispersity index (0.13) established that biosynthesized CuNPs are mainly monodispersed with no agglomeration and homogenous in shape and size distribution. The positive or negative value of zeta potential is a very helpful parameter in the determination of stability of the prepared NPs. Nanoparticles having relatively high zeta potential are more likely to repel each other, which is a very good indicator of the enhancement of stability over a long time. The results of zeta potential measurements showed a value of −23.7 mV which depicts the high stability of the synthesized CuNPs. The result of this experiment agrees well with previous research findings of an earlier work [23].

#### 2.2.4. X-ray Diffraction Analysis

**Figure 3:**
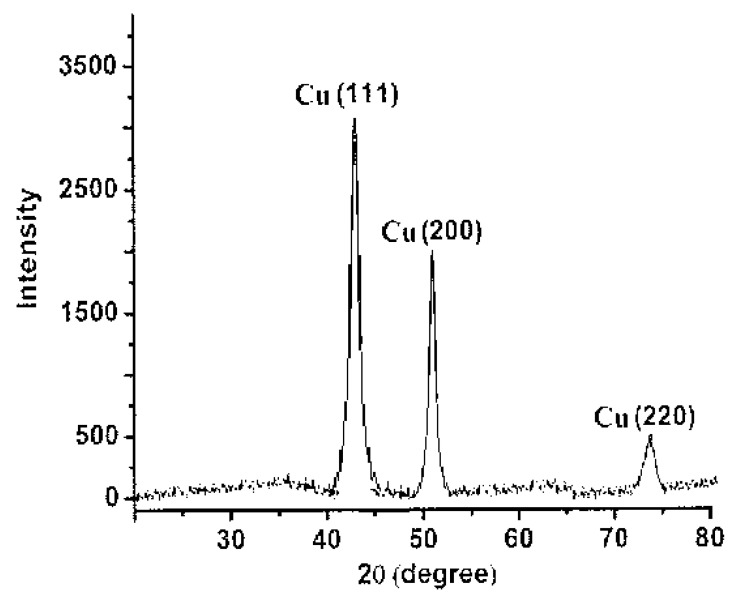
XRD pattern of the TA-mediated CuNPs.

The characteristic crystalline feature of the synthesized CuNPs was confirmed by XRD analysis. The XRD spectrum of CuNPs powder (Figure 3) exhibits a pattern of three peaks observed at 43.4, 50.5, and 74.2°, corresponded to (111), (200), and (220) diffraction planes of metallic copper, which were quite consistent with those of the Joint Committee on Powder Diffraction Standards (JCPDS) for copper (file No. 04-0836). This pattern of XRD spectrum, in good agreement with preceding works [23,24], confirm the formation of a distinctive centered cubic crystalline form of CuNPs.

#### 2.2.5. Scanning Electron Microscopic Analysis

**Figure 4:**
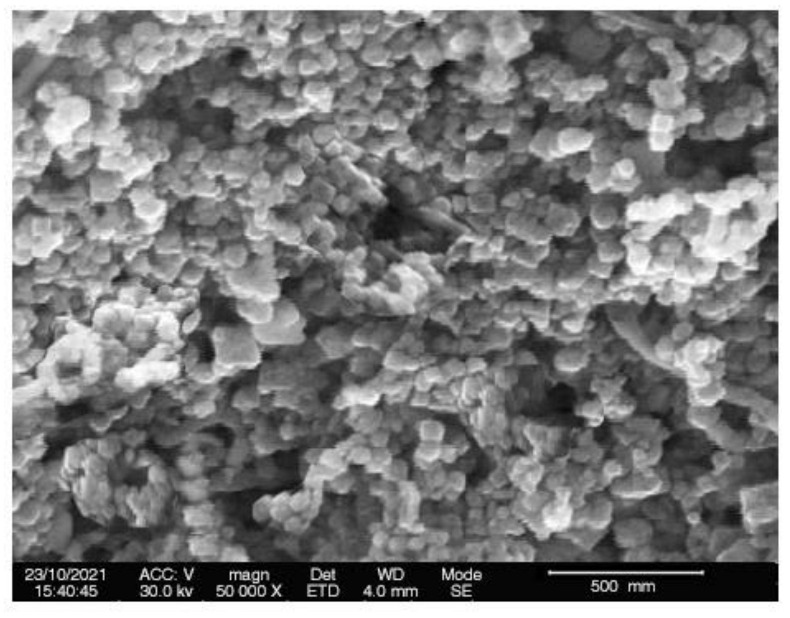
Scanning electron microscopic analysis of the TA-mediated CuNPs.

One of the most extensively utilized techniques for the characterization of nanomaterials and nanostructures is the scanning electron microscopy. The signals generated by electron-sample interactions provide information about the sample, such as the sample’s surface morphology (texture). SEM images (Figure 4) have been employed in this investigation to examine the morphology and size of CuNPs. The particle size of CuNPs (49 ± 3.1 nm) agreed with the results obtained from studying the particle size distribution via Zetasizer as mentioned above. Regarding the shape of CuNPs, from SEM images, it was found to be relatively spherical and homogeneous in appearance [25]. The agglomerated CuNPs shown in the SEM images could be due to coatings of the particles with residual of TA having surface OH, CO, and C-O-C groups.

#### 2.2.6. Antimicrobial Evaluation of TA and Its Mediated CUNPs

Globally, resistance to microorganisms is becoming a foremost public health concern. Antimicrobial drugs are required, particularly against multidrug-resistant microbes. In traditional medicine, tannin-rich plants have been used as a styptic, antidiarrheic, and antiseptic, and have been reported to suppress the growth of a wide range of bacterial and fungal strains [26,27]. Despite the purported health benefits of ellagitannins, insufficient absorption, poor bioavailability, and short retention time may restrict their optimal antimicrobial potential. Therefore, tannin-loaded NPs and hydrogels were also investigated for their antimicrobial properties [28]. Furthermore, various plant extracts rich in tannins have been utilized for the production of metal NPs with considerable antimicrobial activity [11].

In this research, a standard agar well diffusion method was adopted to measure the antimicrobial activity of TA and its mediated CuNPs. TA exhibited varied inhibition areas against *P. aeruginosa* ATCC 27853 (11 ± 0.01 mm), *E. coli* ATCC 25922 (10 ± 0.01 mm), *B. subtilis* NCTC 10400 (9 ± 0.01 mm), *S. aureus* ATCC 25923 (7 ± 0.03 mm), *C. albicans* ATCC 10237 (12 ± 0.03 mm), and *Asp. flavus* ATCC 11267 (9 ± 0.02 mm) (Table 1). The most promising activity was against *P. aeruginosa* ATCC 27853 and *C. albicans* ATCC 10237. The chemical structure of ellagitannins determines their effects on bacterial growth [26]. Previous studies have demonstrated that the presence of free galloyl groups is required for ellagitannins to have antibacterial action [29]. In addition, the digalloyl or trigalloyl groups linked to the glucose core appear to play a role in the antibacterial activity [27]. TA sub-structures, tellimagrandin II and tellimagrandin I [19] have demonstrated promising activity against *helicobacter pylori,* and *H. pylori* and methicillin-resistant *Staphylococcus aureus* (MRSA), respectively [30,31]. Tellimagrandin I suppressed the activity of penicillin-binding protein 2a (PBP2a) in MRSA and reduced β-lactam antibiotic resistance in MRSA [31].

**Figure 5:**
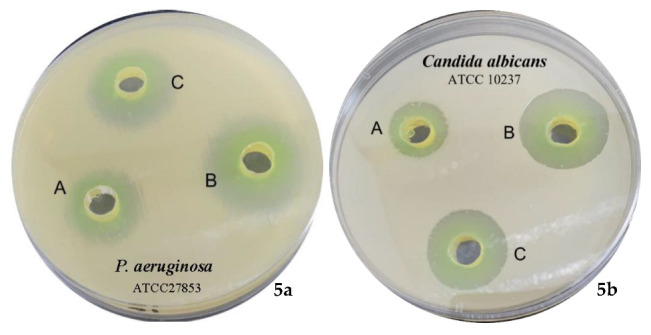
Antibacterial activity of CuNPs against *P. aeruginosa* ATCC 27853 (**5a**) and *C. albicans* ATCC 10237 (**5b**). (A) Tamarixinin A, (B) Formulated CuNPs using tamarixinin A, and (C) Streptomycin, and Fluconazole (10 mg/mL) positive controls.

The activity of copper metal as an antimicrobial agent is well known. It has been registered with the US Environmental Protection Agency as an antimicrobial agent [32]. CuNPs have been shown to interact with bacteria in a unique way [32,33] and have been proven to be effective against a wide range of pathogenic bacteria [34,35]. It has been shown that tannins in a mild alkaline medium mediate the immediate reduction of metal ions into metal NPs with a large capping ability over metal surfaces, primarily through hydroxyl, carbonyl, and C–O–C groups [11,27]. TA in a phosphate buffer (pH 7.4) was thus utilized for the formulation and stabilization of CuNPs. Particle size (44 ± 1.7 nm), polydispersity index (0.13) the high value of zeta potential (−23.7 mV) confirms the formation of CuNPs with high stability [36]. TA mediated CuNPs were assessed for antimicrobial activity against the same strains. In terms of antimicrobial activity, TA mediated CuNPs displayed excellent bactericidal activity in comparison with TA alone (Table 1, Figure 5). The highest levels were observed against *P. aeruginosa* ATCC 27853 and *C. albicans* ATCC 10237, whereas the lowest levels were observed against *S. aureus* ATCC 25923. These results are comparable with previous studies that investigated the antimicrobial properties of Cu metal [37], Cu-loaded nanoparticle [38], and CuNPs mediated by modified polyol method [39] concerning the powerful activity against the fungal strain *C. albicans* (20 ± 0.01 mm) and *A. flavus* (16 ± 0.01 mm). However, TA mediated CuNPs exhibited much higher activity against *P. aeruginosa* (20 ± 0.04 mm) and *E. coli* (18 ± 0.01 mm), comparable activity toward *B. subtilis* (12 ± 0.03), and lower activity toward *S. aureus* (10 ± 0.02 mm) as compared with the reported results of previous investigations [37,38,39]. The fortified activity of TA mediated CuNPs toward *P. aeruginosa* and *E. coli* highlight the presence of a possible modulating mechanism of the TA capping the CuNPs toward these Gram-negative bacteria. Worth mentioning that related ellagitannins from *Tamarix plants* exhibited inhibition of pathogenic pigment production in *P. aeruginosa* [40], which agrees with our results that the TA capping the CuNPs is influencing the antimicrobial activity.

Tannins have been shown to inhibit bacterial growth using various mechanisms, including iron chelation, cell wall inhibition, cell membrane rupture, and inhibition of fatty acid biosynthesis [27]. Thus, TA capping the metal NPs would thus augment their antimicrobial activity toward the Gram-negative bacteria by one or more of these mechanisms, as demonstrated here by the improved antimicrobial activity of the TA mediate CuNPs.

Although CuNPs remain unknown to this day in terms of their exact mechanism of antimicrobial action [6], there is some consensus that several factors contribute to the release of copper ions, their penetration and disruption of cellular membranes and biochemical pathways by chelating cellular enzymes and DNA harm. CuNPs’ inhibitory action may be due to their tiny size and the elevated surface/volume ratio, which enables them to interact with the microbial cell membranes [41]. In addition, CuNPs may inhibit selective permeability by interacting with a deactivated surface protein that transports substances via cytoplasmic membranes [42]. As a consequence of their inactivating effects, free radicals are also produced, which damage DNA and proteins [32]. The sulfur and phosphorous protein moieties in bacterial cell membranes, which are necessary for viability, are attracted to NPs [43]. Nano metals induced a smaller zone of inhibition in Gram-positive bacteria may be due to the protective peptidoglycan layer resisting their action [44].

## 3. Materials and Methods

### 3.1. General Experimental Procedures

Normal-phase high-performance liquid chromatography (NP-HPLC) was conducted on a YMC-Pack SIL A-003 (YMC, Tokyo, Japan) column (4.6 i.d. × 250 mm) developed with *n*-hexane/methanol/tetrahydrofuran/formic acid (55/33/11/1, *v*/*v*) containing oxalic acid (0.45 g/L) at a flow rate of 1.5 mL/min at room temperature. Detection was obtained with a UV detector set at 280 nm. Monitoring of fractionation of gall extract of *T. aphylla* was accomplished on an ODS-A A-303 (YMC, Tokyo, Japan) column (4.6 i.d. × 250 mm) developed using 0.01 M phosphoric acid —0.01 M potassium dihydrogen phosphate-methanol (2:2:1, *v*/*v*) or 0.01 M phosphoric acid —0.01 M potassium dihydrogen phosphate—acetonitrile (42.5:42.5:15, *v*/*v*) at a flow rate of 1 mL/min in an oven set at 40 °C. Measurements of the high-resolution electrospray ionization mass (HRESIMS) spectra were taken on an API-4000 instrument (AB Sciex, Framingham, MA, USA). The solvent used was acetonitrile/water (1:1, *v*/*v*). The ^1^H NMR spectra were recorded on a Varian INOVA AS 600 instrument (Agilent, Santa Clara, CA, USA), 600 × 10^3^ KHz. Chemical shifts are recorded in *δ*_H_ (ppm) values respective to the solvent signal acetone-*d*_6_ (*δ*_H_ 2.04) on the tetramethylsilane scale.

### 3.2. Materials

Copper sulfate was obtained from Sigma Aldrich, USA. Cation adjusted Mueller–Hinton broth was purchased from Thermo Fisher Scientific, Waltham, MA. Mueller–Hinton (MH) agar was obtained from Merck, Darmstadt, Germany, and the Malt extract (ME) agar was from WINLAB, Leicestershire, UK. The chromatographic gels, diaion HP-20, MCI-gel CHP-20P (Mitsubishi Chemical, Tokyo, Japan), Toyopearl HW-40F (TOSOH, Tokyo, Japan), Sephadex LH-20 (GE Healthcare Bio-Science AB, Uppsala, Sweden), and ODS-gel (YMC, Tokyo, Japan) were used. Dimethyl sulfoxide (DMSO) was procured from Sigma-Aldrich Inc., St. Louis, MO, USA.

### 3.3. Purification of TA

The *T*. *aphylla* galls used for this study were gathered from mature trees wildly grown near the coast of the Mediterranean Sea by the side of El-Alamein City, Egypt [15]. TA was purified as reported in our preceding publication [15]. Briefly, the dried galls powder (200 g) were extracted at room temperature in acetone/water [(7:3, *v*/*v*), 3 × 2 L] using a homogenizer. The obtained total extract was concentrated to ~400 mL and then fractionated on a Diaion HP-20 (5.5 i.d. × 63 cm) column with water (3 L), methanol/water (1:1, *v*/*v*, 4.5 L), methanol (3 L), and acetone/water (7:3, *v*/*v*, 2 L), successively. Apart (20 g) of the methanol/water eluate, identified as the tannin fraction as monitored by NP-HPLC, was subjected to a Toyopearl HW-40F (2.2 i.d. × 70 cm) column and eluted with ethanol/water (1:1, 6:4, 7:3, *v*/*v*), ethanol/water (7:3, *v*/*v*)—acetone /water (7:3, *v*/*v*) (9:1, 8:2, 7:3, 6:4, 0:10, *v*/*v*), successively. The Toyopearl ethanol/water (7:3, *v*/*v*) eluate was compiled in six units (U-1—U-6). The U-1 fraction (657 mg) was chromatographed on an MCI-gel CHP-20P (1.1 i.d. × 37 cm) with water, water/methanol (9:1, 8.5:1.5, 8:2, 7.5:2.5, 7:3, 65:35, 1:1, *v*/*v*), and methanol. The water/methanol (8:2, *v*/*v*) eluate furnished pure TA (146 mg).

### 3.4. Spectral Data of TA

TA was obtained as an off-white, amorphous powder, ^1^H NMR [(acetone-*d*_6_—D_2_O, 9:1), 600 × 10^3^ KHz]: *δ*_H_ (α-/β-anomers) 7.63, 7.60 (each s, hellinoyl H-6″), 7.06, 7.05 (each d, *J* = 2.4 Hz, hellinoyl H-6), 6.96, 6.94, 6.90, 6.89 (each s, galloyl (H-2/H-6) × 2), 6.74, 6.73 (each s, hellinoyl H-6′), 6.64, 6.63, 6.628, 6.615 [each s, hexahydroxydiphenoyl (HHDP) H-3 × 2], 6.53, 6.52, 6.48, 6.47 (each s, HHDP H-3′ × 2), 6.03 (1H, d, *J* = 2.4 Hz, hellinoyl H-2), 5.81, t (*J* = 10 Hz, glucose H-3′α), 5.78, d (*J* = 8.4 Hz, glucose H-1α), 5.77, d (*J* = 8.4 Hz, glucose H-1β), 5.74, t (*J* = 10 Hz, glucose H-3), 5.56, t (*J* = 9.6 Hz, glucose H-3′β), 5.51, d (*J* = 3.6 Hz, glucose H-1′α), 5.42, dd (*J* = 8.4, 9.9 Hz, glucose H-2β), 5.39, dd (*J* = 8.4, 9.9 Hz, glucose H-2α), 5.29, dd (*J* = 6.6, 13.2 Hz, glucose H-6), 5.26, dd (*J* = 7.8, 9.6 Hz, glucose H-2′β), 5.26, dd (*J* = 6.6, 13.2 Hz, glucose H-6′β), 5.24, dd (*J* = 3.6, 10 Hz, glucose H-2′α), 5.24, dd (*J* = 6.6,13.2 Hz, glucose H-6′α), 5.15, d (*J* = 7.8 Hz, glucose H-1′β), 5.09, t (*J* = 10 Hz, glucose H-4), 5.06, t (*J* = 9.6 Hz, glucose H-4′β), 5.05, t (*J* = 10 Hz, glucose H-4′α), 4.67, ddd (*J* = 1.2, 6.6, 9.9 Hz, glucose H-5′α), 4.42, ddd (*J* = 1.2, 6.6, 9.9 Hz, glucose H-5), 4.26, dd (*J* = 6.6, 9.6 Hz, glucose H-5′β), 3.88, d (*J* = 13.2 Hz, glucose H-6′β), 3.85, dd (*J* = 1.2, 13.2 Hz, glucose, H-6), 3.78, d (*J* = 1.2, 13.2 Hz, glucose, H-6α). HRESIMS *m*/*z* 1743 [M + Na]^+^ (C_75_H_52_O_48_) [19].

### 3.5. Green Synthesis of CuNPs

Copper sulfate has been used as an initiator substance in the preparation of CuNPs, while TA has been used as a reductant and coating agent. A solution of copper sulfate 5 mM was prepared by dissolving the exact amount of copper sulfate in deionized water. Copper sulfate solution is characterized by its green color. To the above solution, TA (0.001% in phosphate buffer, 0.01 M phosphoric acid—0.01 M potassium dihydrogen phosphate, pH 7.4) was added in the reaction vessel in a 1:2 ratio (*v*/*v*). The reaction vessel was kept at a water bath shaker at 50 °C for 2 h. Then the mixture was allowed to cool for about 12 h at room temperature. After that, the obtained mixture was centrifuged at 1 × 10^4^ rpm for 10 min and the residue was rinsed three times with water and redispersed in water for further studies.

### 3.6. Characterization of TA-Mediated CuNPs

#### 3.6.1. UV-Visible Spectroscopy

The production and stability of CuNPs in reaction vessels were both monitored using UV-visible spectroscopy. The wavelength was set at 400–800 nm, and deionized water was used as the reference standard.

#### 3.6.2. Particle Size, Zeta Potential, and Polydispersity Index Evaluation

Zetasizer nano series (Malvern Instruments, Malvern, UK) was used to measure the particle size of the CuNPs dispersed in water. The homogeneity and stability of the synthesized CuNPs were assessed by measuring polydispersity index and zeta potential respectively.

#### 3.6.3. X-ray Diffraction Analysis

To confirm the crystalline nature of the synthesized CuNPs, XRD analysis was performed using Ultima IV diffractometer (Rigaku, Japan). The sample was examined using Cukα radiation (λ = 0.154056 nm), generated at 40 × 10^3^ V, 40 mA and a receiving hole of 0.3 mm. The spectra were observed over the 2θ range of 20–80° with an angular increment of 1°/ 2 min and a scanning speed of 1.0 s.

#### 3.6.4. Scanning Electron Microscopic (SEM) Analysis

SEM (Carl Zeiss EVO LS10, Cambridge, UK) was used to identify the surface morphology of the biosynthesized CuNPs.

### 3.7. Antimicrobial Evaluation

#### 3.7.1. Culture and Microbial Growth

A cation adjusted Mueller–Hinton broth (CAMH) was used to grow the tested bacterial strains, whereas the fungal strains were grown to the mid-log phase using Sabauraud dextrose broth. Bacterial and fungal suspensions were measured at 625 nm using a spectrophotometer to calculate an absorbance of 0.12 (1 × 10^8^ CFU/mL). A bacterial concentration of 1 × 10^6^ CFU/mL was obtained by diluting the suspension (1:100) in CAMH broth. The tested bacterial strains were two Gram-positive (*B. subtilis* NCTC 10400 and *S. aureus* ATCC 25923) and two Gram-negative (*P. aeruginosa* ATCC 27853 and *E. coli* ATCC 25922), while *C. albicans* ATCC 10237 and *A. Flavus* as unicellular bacteria and fungi, respectively. The bacterial and fungal strains were provided by Biotechnology Lab, Pharmaceutics Department, Faculty of Pharmacy, King Saud University.

#### 3.7.2. Antimicrobial Assay of TA and TA-Mediated CuNPs

Both TA and the synthesized CuNPs formula were evaluated for antimicrobial activity via standard agar well-diffusion method contrary to Gram-negative bacteria, Gram-positive bacteria, and fungi [33]. The MH agar was utilized for bacteria and the ME agar was utilized for fungi. Bacterial meadows were prepared for each test organism using an aliquot of 100 μL bacterial suspension. The adjusted microbial suspension was spread on MH agar for bacteria and ME agar for fungi and allowed to dry completely. In the agar plate, a sterilized stainless steel Cork borer was applied to create 6 mm wells in diameter. The tested well was loaded with CuNPs solution (50 μL of 1 mg/mL concentrations) and TA (50 μL of 1 mg/mL concentrations) using a calibrated pipette. Bacteria were incubated at 35 ± 1 °C for 24 h and fungi at 25 ± 1 °C for 5 days. After the incubation period, they were inspected for inhibition zones. The inhibition diameter was measured for each organism, and the mean value in millimeters was recorded. Positive controls for antibacterial and antifungal activities were streptomycin and fluconazole (10 mg/mL), respectively. The negative control was DMSO. The experiment was repeated twice, and the mean diameter of the NPs was calculated which reflected their inhibitory nature.

## 4. Conclusions

Tamarixinin A ellagitannin was utilized for a facile formulation of CuNPs with small and stable particle sizes. CuNPs formulated utilizing TA marking the first research implicating pure ellagitannin in nanoparticle synthesis. CuNPs mediated by TA could be used to tackle infections caused by *C. albicans*, *A. flavus*, and *P. aeruginosa*, as well as to decontaminate medical devices and related objects. Thus, the considerable antibacterial action of TA-mediated CuNPs against *P. aeruginosa* shows that TA is actively involved, which is an encouraging discovery.

## Figures and Tables

**Table 1:** In vitro antimicrobial activity (average diameters of inhibition zones) of TA and its mediated CuNPs against different bacterial and fungal strains.

	Inhibition Zone Diameter (mm)					
	Gram-Positive Bacteria		Gram-Negative Bacteria		Fungi	
	*B. subtilis* NCTC 10400	*S. aureus* ATCC 25923	*E. coli* ATCC 25922	*P. aeruginosa* ATCC 27853	*C. albicans* ATCC 10237	*Asp. flavus* ATCC 11267
Tamarixinin A	9 ± 0.01	7 ± 0.03	10 ± 0.01	11 ± 0.01	12 ± 0.03	9 ± 0.02
CuNPs	12 ± 0.03	10 ± 0.02	18 ± 0.01	20 ± 0.04	20 ± 0.01	16 ± 0.01
Streptomycin	16 ± 0.00	14 ± 0.01	16 ± 0.01	16 ± 0.02	ND	ND
Ketoconazole	ND	ND	ND	ND	18 ± 0.02	17 ± 0.00
DMSO	No	No	No	No	No	No

Values are Mean ± SD, ND means not determined, No means no inhibition zone.

## Data Availability

The data is contained within the article and Supplementary Materials.

## Supplementary Materials

The following are available online at https://www.mdpi.com/article/10.3390/ph15020216/s1, Figure S1: ^1^H NMR spectrum of tamarixinin A [600 MHz, (acetone-*d*_6_ + D_2_O, 9:1), 27 °C]. Figure S2: Expanded ^1^H NMR spectrum of tamarixinin A [600 × 10^3^ KHz, (acetone-*d*_6_ + D_2_O, 9:1), 27 °C]. Figure S3: HRESIMS of tamarixinin A.
