# Supplementary material for: Design, Characterization, and Antimicrobial Evaluation of Copper Nanoparticles Utilizing Tamarixinin a Ellagitannin from Galls of Tamarix aphylla
Source: Pharmaceuticals (Basel). 2022 Feb 11;15(2):216. doi: 10.3390/ph15020216 (PMC8874630; doi:10.3390/ph15020216)
Supplement: Supplementary file 1 [file pharmaceuticals-15-00216-s001.zip › pharmaceuticals-1587494-supplementary.pdf]

SUPPLEMENTARY DATA

Design, Characterization, and Antimicrobial Evaluation of Copper Nanoparticles Utilizing Tamarixinin A Ellagitannin from Galls of *Tamarix aphylla*

Mohamed A. A. Orabi [1,*], Mounir M. Salem-Bekhit [2,3], Ehab I. Taha [4], El-Shaymaa Abdel-Sattar [5], Omaish Salman Alqahtani [1], Fakhria A. Al-Joufi [6], Basel A. Abdel-Wahab [7], Ali Mohamed Alshabi [8], Hamad S. Alyami [9,*], Javed Ahmad [9] and Tsutomu Hatano [10]

[1] Department of Pharmacognosy, College of Pharmacy, Najran University, Najran 1988, Saudi Arabia; osalqahtani@nu.edu.sa

[2] Kyyali Chair for Pharmaceutical Industry, Department of Pharmaceutics, College of Pharmacy,
King Saud University, Riyadh 11451, Saudi Arabia; mounirmsalem@yahoo.com

[3] Microbiology and Immunology Department, Faculty of Pharmacy, Al-Azhar University, Cairo 11884, Egypt

[4] Department of Pharmaceutics, College of Pharmacy, King Saud University, P.O. Box 2457, Riyadh 11451, Saudi Arabia; eelbadawi@ksu.edu.sa

[5] Department of Microbiology and Immunology, Faculty of Pharmacy, South Valley University, 83523 Qena, Egypt; elshaymaa_a_m@svu.edu.eg

[6] Department of Pharmacology, College of Pharmacy, Jouf University, Al-Jouf 72341, Saudi Arabia; faaljoufi@ju.edu.sa

[7] Department of Pharmacology, College of Pharmacy, Najran University, Najran 1988, Saudi Arabia;
babdelnaem@nu.edu.sa

[8] Department of Clinical Pharmacy, College of Pharmacy, Najran University, Najran 1988, Saudi Arabia;
amalshabi@nu.edu.sa

[9] Department of Pharmaceutics, College of Pharmacy, Najran University, Najran 1988, Saudi Arabia;
jaahmed@nu.edu.sa

[10] Graduate School of Medicine, Dentistry and Pharmaceutical Sciences, Okayama University, Tsushima,
Okayama 700-8530, Japan; hsalmukalas@nu.edu.sa

* Correspondence: maorabi@nu.edu.sa (M.A.A.O.); hsalmukalas@nu.edu.sa (H.S.A.); Tel.: +966-557398835 (M.A.A.O.); +966-500095255 (H.S.A.)

List of contents

Content	Page
**Figure S1.** ^{1}H NMR spectrum of tamarixinin A [600 × 10^3 KHz, (acetone-d_6 + D_2O, 9:1), 27°C].	S3
**Figure S2.** Expanded ^{1}H NMR spectrum of tamarixinin A [600 × 10^3 KHz, (acetone-d_6 + D_2O, 9:1), 27°C].	S4
**Figure S3.** HRESIMS of tamarixinin A.	S5

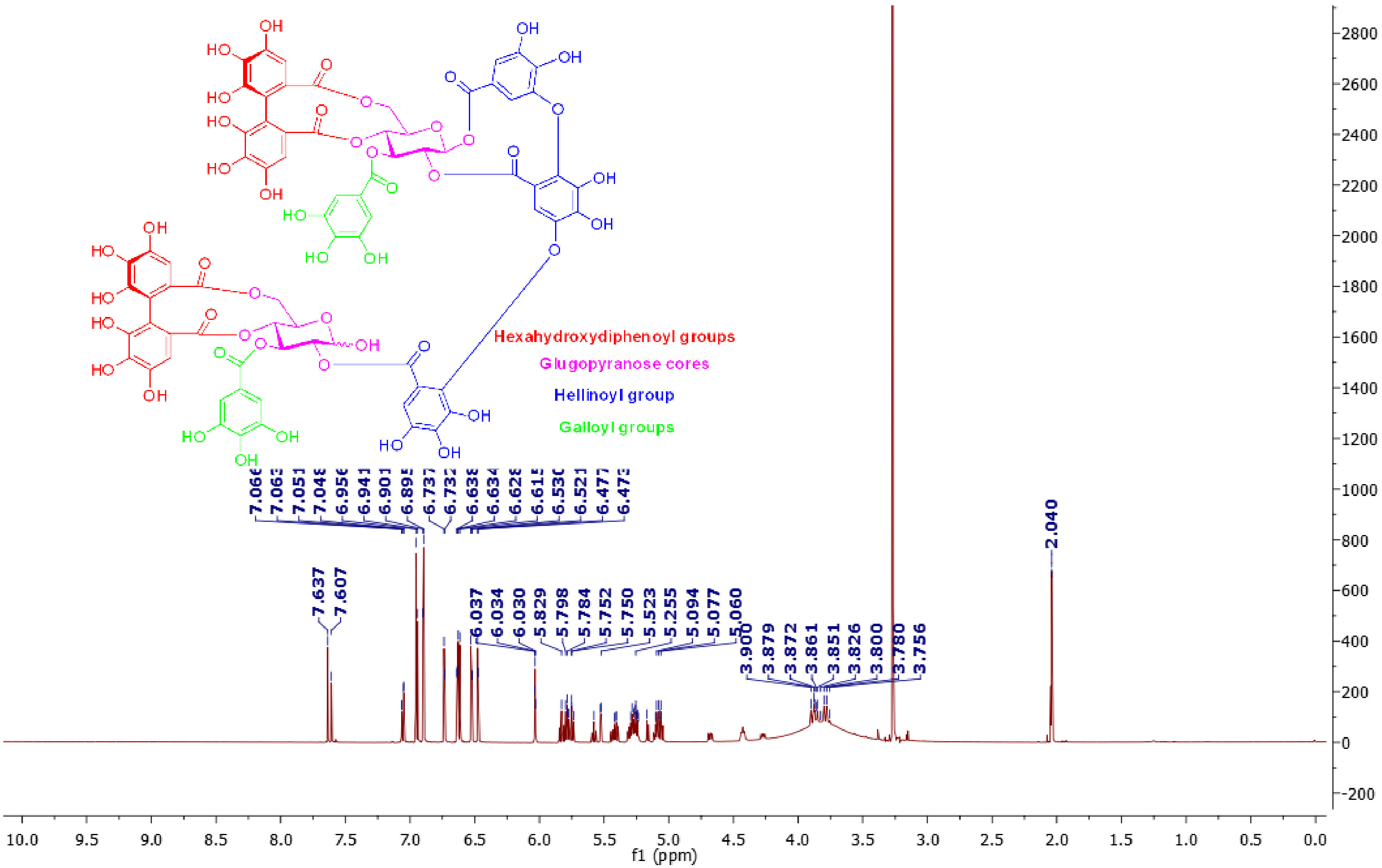

Figure S1. ^{1}H NMR spectrum of tamarixinin A [600 × 10^3 KHz, (acetone-d_6 + D_2O, 9:1), 27 °C].

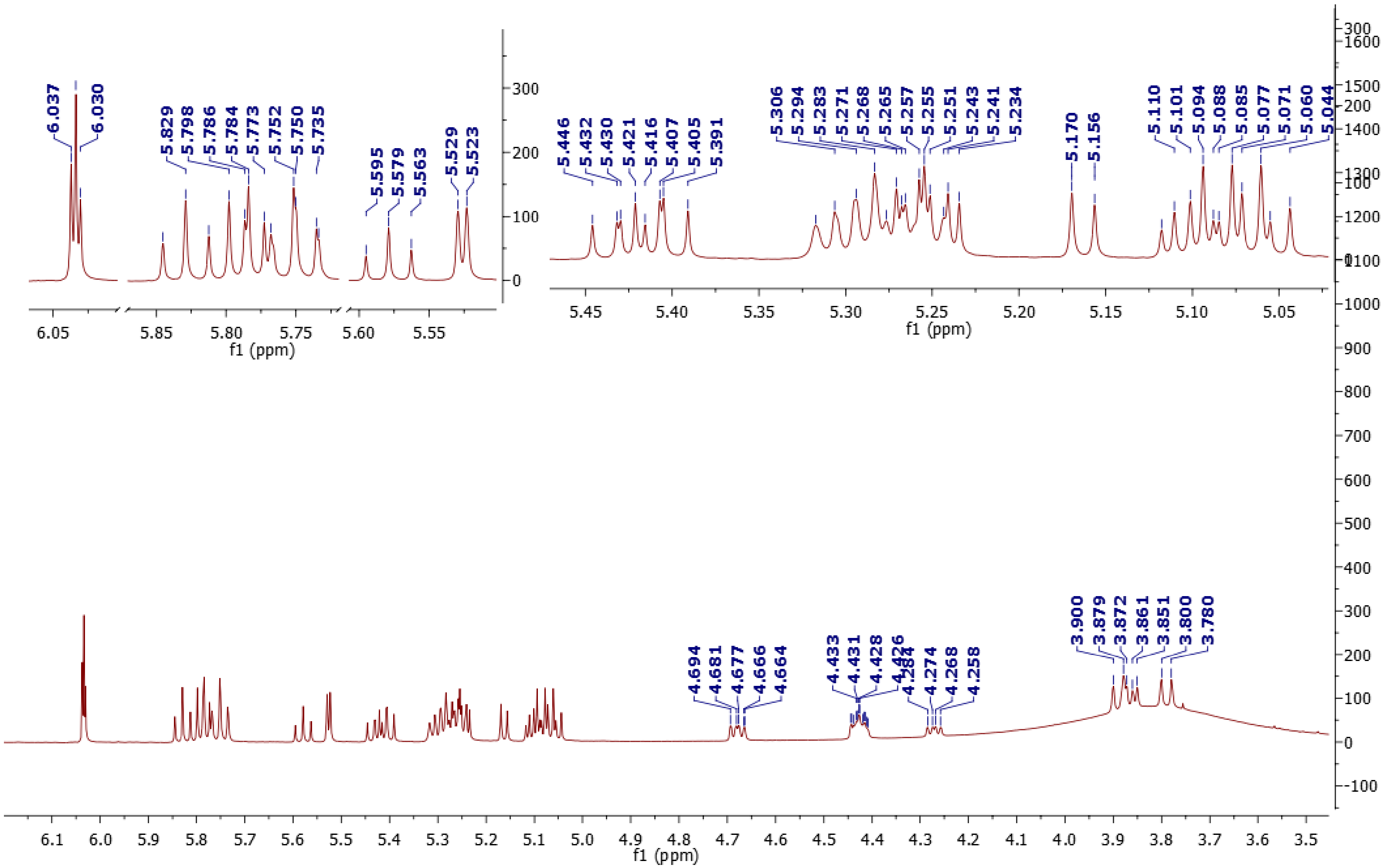

Figure S2. Expanded ^{1}H NMR spectrum of tamarixinin A [600 × 10^3 KHz, (acetone-d_6 + D_2O, 9:1), 27 °C].

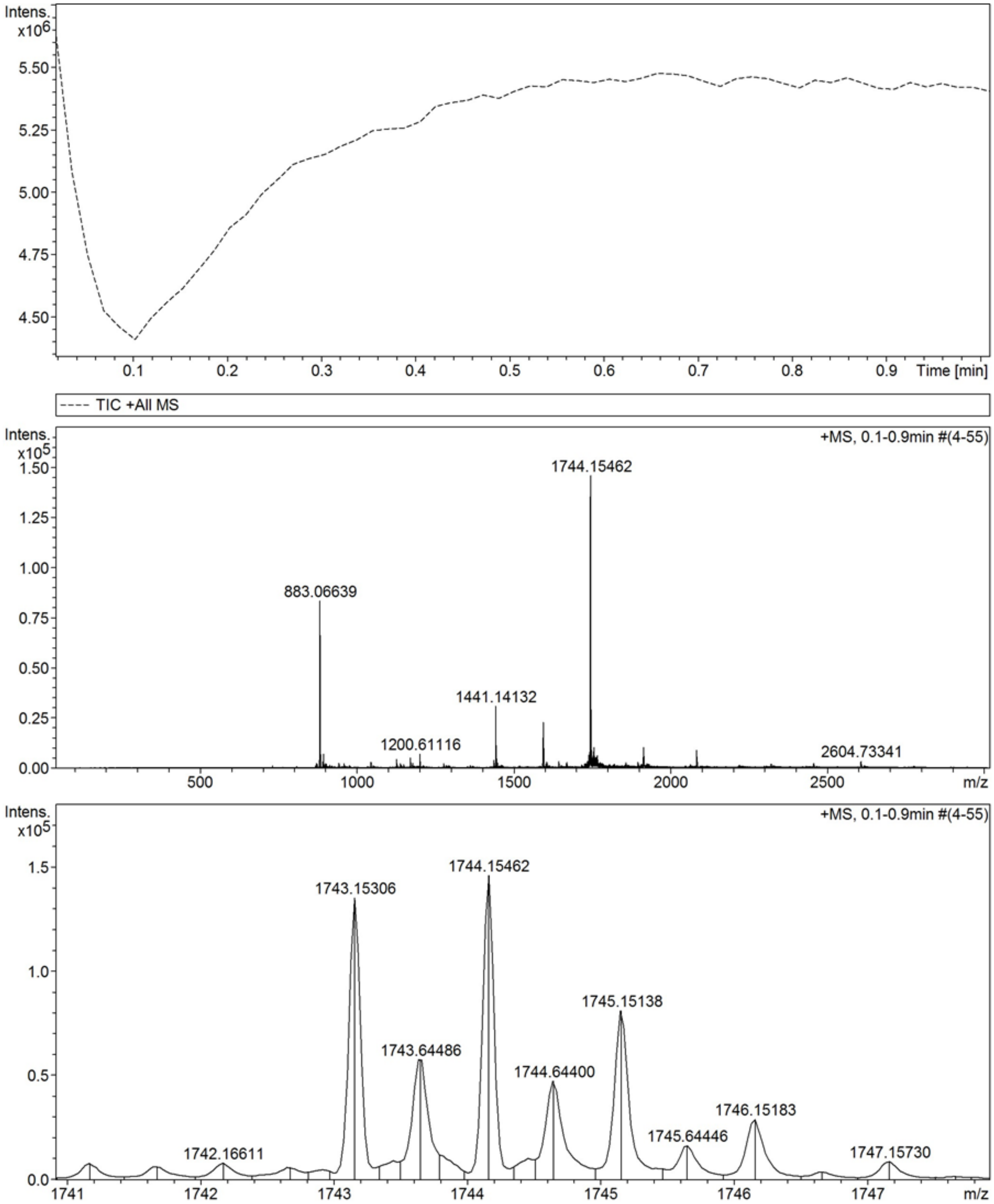

Figure S3. HRESIMS of tamarixinin A.
